# The Diagnosis and Treatment of Dietetic Albuminuria

**Published:** 1898-10

**Authors:** J. Howe Adams

**Affiliations:** Overbrook, Pa.


### Southern Medical Record.

A MONTHLY JOURNAL OF MEDICINE AND SURGERY.

VOL. XXVIII. ATLANTA, GA., OCTOBER, 1898. NO. 10.

### Original Articles.

### THE DIAGNOSIS AND TREATMENT OF DIETETIC ALBUMINURIA.

By J. HOWE ADAMS, M. D., Overbrook, Pa.

Considerable confusion exists in many quarters as to the meaning and limitation of the term "dietetic albuminuria," its symptoms, its pathology and its treatment. True dietetic albuminuria is that form in which the albumin is found in the urine only after certain kinds of food have been ingested, being taken as a rule in immoderate amounts, such as eggs, cheese or pastry. Consequently this condition is not constantly present but occurs at special periods, such as after breakfast or a noonday meal. It can only follow the taking of food, and as a rule, it follows this action very promptly; it is unattended by any other sign of disease, tube casts being totally absent.

It is a very important factor in the diagnosis to remember that albumin is never found in the urine before breakfast, and there is lacking all signs of disorder of the nervous system as is found commonly in other forms of kidney trouble. The urine shows a high specific gravity; in fact, many observers lay great stress on this point, claiming that this condition is invariable in these cases. This point of high specific gravity is one of great importance in kidney trouble; except in very acute cases of Bright's disease or in those advanced cases where there is a diminution of the solid matter in the urine which is counterbalanced by the decrease in the water excreted, thus raising the specific gravity, the presence of high specific gravity in any urine is fair presumptive evidence that the urine is not from a case of Bright's disease. It is well to remember that high specific gravity in the urine is often associated with what might be called "harmless albuminuria."

The absence of albumin in the early morning urine in dietetic albuminuria becomes a matter of great importance in many instances, even outside strictly medical matters; for example, Dr. Pavy of London relates a case occurring in his practice in which a young lad seeking a position in the English civil service was rejected on the ground of the presence of albumin in his urine; his medical adviser thought of the possibility of its being this form of albuminuria and found that his urine passed on rising was perfectly normal in its constituents. This statement applies in this country with great force to candidates for life insurance.

As the symptoms of dietetic albuminuria are characteristic, it would seem easy to distinguish it from other expressions of disease, but such is not always the case. The albuminuria following severe exercise is occasionally mistaken for this form; its diagnostic point is the fact that it occurs only after strenuous exertion, while there is an absence of all other symptoms both as regards the nervous system and the digestive state. The severe exertion does not produce albumin in a distinctive or striking manner, and as soon as the strain is over the abnormal ingredient at once disappears. It is much more difficult to distinguish between true dietetic albuminuria and that due to mal-assimilation; in fact, these forms are generally confused. Possibly the difference is only one of degree, but in that form due to excessive uric acid formation, the symptoms are much more marked than in the pure dietetic form. In the uric acid variety the patient suffers from indigestion, flatulence and constipation; he is very nervous; mentally he is greatly depressed about himself and he is frequently annoyed by an irritable bladder. Albumin is found in the urine, frequently being most marked in the afternoon evacuations. Occasionally a few epithelial casts may be found; the urine is strongly acid with a specific gravity rarely below 1022 and with deposits of quantities of "brick-dust." This uric acid mal-assimilation may become so marked that it will be confused with the ordinary form of Bright's disease, especially when the patient exhibits some body waste, indisposition to either mental or physical exertion, and is easily fatigued with the production of frequent headaches and vertigo. But to distinguish this mal-assimilative condition from true Bright we soon see that the hypertrophies and other cardiac lesions of Bright's disease are wanting; there is no dropsy or eye lesions and the actual character of the urine is most significant. It is in the urine that the diagnosis between uric acid trouble and the other varieties must be made, after all; first of all there is the high specific in uric acid conditions in a urine that is about normal in amount or a little scantier than normal. The specific gravity generally ranges high, from 1022 to even as high as 1036. The urine on standing deposits urates, even uric acid and frequently mucus; or in the deposits in the place of the urates, are often found crystals of the oxalate of calcium, for the uric acid condition does not necessarily show itself in the presence of urates in the urine. The solid matters are increased but the salts vary but little, although the chlorides are increased and not diminished, as they are in contracting kidney.

When it comes to the differential diagnosis between true dietetic albuminuria and that due to uric excess, we find that in the latter condition the amount of albumin is more regularly present in the urine although it varies with the time of day, being more pronounced after breakfast while it is frequently absent after the evening meal. But here, as in other cases of so-called intermittent albuminuria, there is this fallacy to be remembered—the albumin is not really absent, but is only greatly diminished. A fine test, such as the use of acetic acid to acidulate the urine, and then the use of Tanret's test may bring it out. Casts are scanty or absent in uric acid conditions as well as in the true dietetic form; when present they are hyaline or epithelial in character, rarely granular, never fatty.

The pathological condition which gives rise to the album of uric acid is essentially a congestion of the kidney with slight local inflammation in the vascular cortex from the irritating effects of secreting increased amounts of ill-formed or broken-down tissue in the form of urates urea or those imperfectly oxidized as oxalates. In other words, the kidneys are not the primary cause of trouble, but simply the kidneys suffer because they are compelled to throw off irritating waste-products. Anything that lowers vitality and disturbs the nervous system may increase the faulty assimilation and, with it, the irritation and congestion which produce the albuminuria. All these symptoms are more marked than those occurring in the true dietetic form, so that a careful examination should show a distinction.

When it comes to the important question of treatment of these forms of albuminuria, we must bear in mind that we are dealing with special diseases in which mal-assimulation is the main element and the kidney affection only a conspicuous expression. The treatment must not be therefore purely that of Bright's disease, as we meet it commonly, but largely that of the underlying condition, a state that borders on lithæmia. The lines of treatment in these cases are therefore parallel. It is essential to lessen the work on the kidneys as in ordinary forms of Bright's disease by calling into play the various emunctories. It is quite as essential to lessen the load on the kidneys, for it is possible for this condition to pass into true organic affection of the kidneys, although considering the force of the irritating agent, this occurs far less frequently than would be supposed at first thought. When it does happen, it is brought about by the constant irritation, leading to slow fibroid changes and gradually developing interstitial nephritis. In the first place, to keep the kidneys flushed, the free use of pure water should be urged, plain or aerated; and the mild diuretic waters, such as Poland, Saratoga Vichy or Vichy, are always of service. Drinking hot water at bed-time often has a most happy diuretic effect which makes it doubly serviceable, for these products are largely excreted in the morning hours. Alcoholic drinks should be avoided, or, if used, restricted to light non-acid wines; no beer should be permitted.

To further our purposes still more, it is well to advocate the use of baths, not too cold, followed by systematic skin friction; while the value of open-air exercise is frequently most marked. Cold douches to the spine are recommended by some authorities, especially in the true dietetic form of albuminuria. Among laxatives, Rochelle salt, cream of tartar and phosphate of soda are the most valued. Mercury before the saline is often of excellent effect; also is iron, especially in the form of the tartrate of iron and potassium. Nitro-muriatic acid is a stand-by in many of these cases. The heart must not be forgotten in these cases, for the poorly oxidized elements circulating in the blood and the general mal-nutrition and the lowered nerve-force disturb its tone; hence any lack of steadiness it exhibits should be met by digitalis or strychnine. The inhalation of oxygen has been suggested, but its therapeutic value has not been settled.

But the most important indication in these cases is in the matter of diet; by paying close attention to the food ingested, the labor of the kidneys can be much lessened while the character of the blood can be greatly improved. Vegetables, especially the green vegetables and fruits, are freely allowed; tea, coffee and cocoa are sanctioned, if sweetened but slightly, as are small quantities of oatmeal, buckwheat, corn cakes, rice, bread and butter, also oysters and fish. The white meat of poultry and game can be eaten in moderation, but the meats containing much nitrogen, such as mutton and beef, should be tabooed. In my experience, however, when the patient indulges in considerable exercise, especially in young men, meats may be eaten in careful amounts without damage. Milk does not seem to be of especial value in these cases; in fact, there are a number of cases on record where a strictly vegetable diet effected permanent cures after exclusive milk diet had left the urine still albuminous. Eggs need not be forbidden, but should be eaten in moderation and the yellow portion alone selected for that purpose. It is well to be strict as to sugars, for most commonly they aggravate the symptoms; hence beets and rhubarb should also be forbidden. Salt, on the other hand, should be freely given.

As to the prognosis, it is favorable when we can make the treatment effective. Thus we have a form of albuminuria which has been rescued from the list of Bright's disease; its clinical history impresses on us that in the examination of the urine it is not sufficient to make the ordinary tests for albumin, but the investigation must be pushed further. If the urine is examined in a routine way without regard to these points, the patient will be often condemned when his condition does not justify it at all.

---

Ocular Affections in the Early Stages of Syphilis.—Wilbrand and Staelin examined the eyes of 200 patients in the early period of syphilis. They found disease of the eyelids in 20 per cent. of the cases, viz.: Hyperemia ciliorum, edema and redness of lids, blepharitis ciliaris, slight ptosis, and the like.

The conjunctiva was hyperemic in 17 per cent., abnormally pale in 5 per cent. In 10 per cent. there were papules on the mucosa.

The cornea showed unilateral parenchymatous inflammation in one case; the sclera in one case showed bilateral episcieritis.

There was iritis in 5 per cent. complicated with choroiditis in 2 per cent.

The optic nerve was affected in 14 per cent. of cases, the affections comprising hyperemia, neuritis, retrobulbar neuritis, and neuro-retinitis.

There was moderate contraction of the visual field in 36 per cent., due mostly to the disturbance of the general condition.

---

The Mauser Bullet.—In a paper read before the N. Y. Academy of Medicine, Dr. Wm. D. Bell (*Medical Record*) stated that at a greater range than 200 yards the Mauser bullet gave a small wound of entrance and only a slightly larger wound of exit. If the bone was shot through at shorter range there was always some comminution. In several instances the Mauser bullet passed through the skull and out again without producing any apparent injury to the brain, and the men returned to duty in two or three days.

